# RNA degradation patterns in cardiac tissues kept at different time intervals and temperatures before RNA sequencing

**DOI:** 10.1371/journal.pone.0323786

**Published:** 2025-05-15

**Authors:** Stine Bøttcher Jacobsen, Jacob Tfelt-Hansen, Morten Holdgaard Smerup, Niels Morling, Jeppe Dyrberg Andersen

**Affiliations:** 1 Section of Forensic Genetics, Department of Forensic Medicine, Faculty of Health and Medical Sciences, University of Copenhagen, Copenhagen, Denmark; 2 Department of Cardiology, Rigshospitalet, Copenhagen University Hospital, Copenhagen, Denmark; 3 Department of Cardiothoracic Surgery, Rigshospitalet, Copenhagen University Hospital, Copenhagen, Denmark; University of Catania, ITALY

## Abstract

Vast repositories of tissues are available in biobanks worldwide. For these tissues to be used for molecular investigations, such as gene expression analysis, it is important to understand the limitations of pre-analytical variables. Storage times and temperature may influence the integrity of the tissue and thereby affect the results of gene expression analyses. To evaluate the effect of storage time at different temperatures, we performed whole transcriptome sequencing of human right atrial appendage tissues stored at either 4°C or 22°C for zero, one, seven, 14, or 28 days. We observed a temperature-dependent RNA degradation with time, as RNA was more stable at 4°C than 22°C. We found that nuclear protein-coding RNAs appear to degrade faster than RNAs encoded by the mitochondrial genome. The global gene expression profiles were relatively stable for up to 24 hours. However, more than seven days of storage induced widespread changes in the gene expression profiles. These changes may, though, be counteracted by including the RNA integrity number as a covariate in the differential expression analyses. We recommend storing tissues at temperatures below 4°C or limiting storage time at temperatures above 4°C in order to produce reliable gene expression profiles.

## Introduction

As high-throughput sequencing technologies become more widely applied, the demand for high-quality tissue specimens grows. Especially with investigations of tissue-specific patterns, the availability of relevant tissue constitutes a significant limitation. Therefore, it is essential to maintain the integrity of tissues to ensure consistency and reproducibility of experimental results. The effects of preanalytical variables, such as tissue collection, handling, and storage, must, hence, be considered to avoid systematic biases [1–4].

RNA degradation due to prolonged tissue storage has been examined with relatively short time intervals. However, the results are conflicting. Multiple studies have reported that the integrity of RNA remains stable for up to 16 hours [5–10], whereas others have shown that the integrity of RNA decreases less than one hour after tissue collection [11–13]. However, to our knowledge, limited information is available on RNA integrity in tissues stored for > 24 hours after tissue collection.

The gradual degradation of RNA with time seems to be temperature-dependent, as RNA is best preserved at cold temperatures [14]. Additionally, it has been suggested that different RNA types have distinct degradation patterns, which may affect the conclusions of gene expression investigations [11,15].

Even with relatively intact RNA, it is important to evaluate the effect of prolonged tissue storage on gene expression profiles. The storage time has been shown to affect gene expression patterns examined with qPCR, even in cases with intact RNA [14,16]. The consequences of RNA degradation when performing polyA-enriched RNA sequencing have been assessed [15,17]. However, a knowledge gap remains when performing whole transcriptome sequencing (WTS) from degraded RNA. Such evaluations may be relevant in situations where surgically resected tissues cannot be stored optimally immediately following excision, such as emergency settings and procedures in facilities with limited opportunities for tissue storage. In these situations, it is of great value to know if tissues are still suitable for gene expression investigations after, e.g., prolonged transportation. Gene expression investigations in a forensic setting with prolonged post-mortem intervals (PMI) may also be challenging. The effect of PMI < 8 hours on human RNA levels has been investigated [15]. However, RNA levels measured in post-mortem tissue samples are likely affected by both biological responses to organism death and RNA degradation occurring as a consequence of environmental factors, whereby degradation patterns may be complex.

In this study, we investigated the changes in RNA integrity and global gene expression patterns in cardiac tissue stored for zero, one, seven, 14, and 28 days before RNA extraction. In addition, we evaluated the effect of temperature by storing the tissues at both 4°C and room temperature (22°C). By immediately acquiring the tissue following excision from the patient, we could control the environmental variables, thereby enabling a precise investigation of RNA degradation patterns induced by storage time and temperature. A paired setup accounted for inter-individual differences in global gene expression profiles. We performed WTS to assess the effects on both coding and non-coding transcripts.

## Materials and methods

### Ethics statement

The study conformed to the Declaration of Helsinki and was approved by the Committees of Health Research Ethics in the Capital Region of Denmark (H-20039524). The project is registered at the University of Copenhagen’s joint records of processing of personal data in research projects and biobanks (514-0528/20-3000), and it complies with the rules of the General Data Protection Regulation (Regulation (EU) 2016/679). Informed written consent was collected from all individuals. Patient material and data were pseudonymised.

### Study population and tissue collection

**Fig 1:**
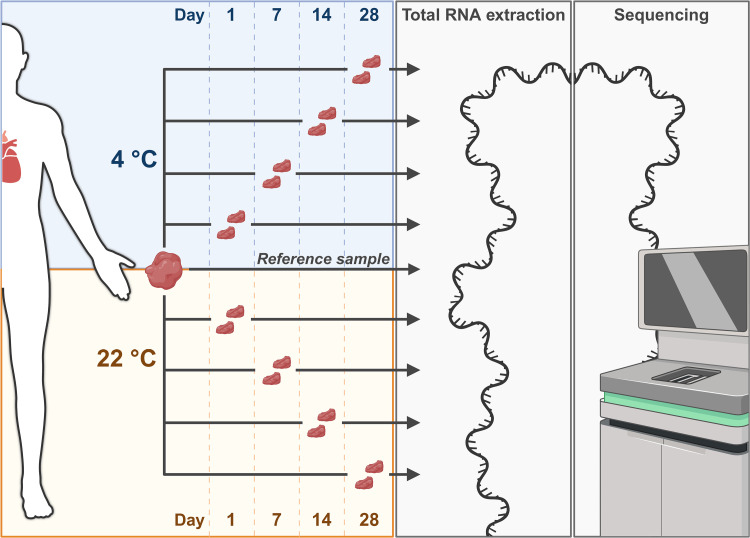
Workflow. Right atrial appendage tissue was collected from nine patients. Tissues were kept at varying temperatures (4°C or 22°C) and time intervals (zero (reference), one, seven, 14, or 28 days) before RNA extraction and subsequent sequencing. All combinations of temperatures and times were investigated in duplicate. Created with Biorender.com.

Human right atrial appendage (RAA) tissue was collected from nine patients who underwent scheduled cardiac surgery at the Department of Cardiothoracic Surgery, Rigshospitalet, Copenhagen, Denmark in the period 6 January 2022 to 16 February 2022 (S1 Table). All RAA tissue samples were divided into 18 pieces (~3 × 3 × 3 mm), where two were subjected to RNA extraction immediately after tissue collection (day zero – reference sample) (Fig 1). The remaining 16 pieces were stored in 2 ml Eppendorf tubes, placed in a cardboard box, and kept at the following time intervals and temperatures: one, seven, 14, and 28 days at either 4°C or room temperature (22°C) (Fig 1). For each combination of time and temperature, two RAA pieces were used for RNA sequencing to enable duplicate investigations. To prevent drying of the tissues, 10 µl isotonic phosphate buffered saline (PBS) was added to the samples.

### RNA extraction

Total RNA was extracted using the RNeasy Fibrous Tissue Mini Kit (Qiagen, Germany) following the manufacturer’s instructions. Up to 30 mg of tissue was used as input. Before RNA extraction, the tissues were homogenised for 2 x 2 min at 20 Hz using a TissueLyser II (Qiagen, Germany). To remove genomic DNA, on-column DNase treatment was performed.

The quality of the RNA was assessed using the Bioanalyzer RNA 6000 Pico Assay Kit with the 2100 Bioanalyzer system (Agilent Technologies, Inc., USA). The RNA integrity number (RIN) and the percentage of RNA fragments >200 nucleotides (DV200) were calculated from the electropherograms. The 2100 Bioanalyzer system (Agilent Technologies, Inc., USA) could not calculate the RIN for one RNA extract. The Qubit RNA HS Assay (Thermo Fisher Scientific, USA) was used to assess the quantity of the extracted RNA.

### Whole transcriptome sequencing

Library preparation was performed using the SMARTer^®^ Stranded Total RNA-Seq Kit v3 – Pico Input Mammalian (Takara Bio Europe, France) following the manufacturer’s recommendations. A total of 10 ng RNA was used as input for library preparation. Before cDNA synthesis, the RNA was fragmented according to the manufacturer’s instructions. Subsequently, cDNA synthesis was performed using random N6 primers to ensure cDNA synthesis from all RNA fragments, and barcoded adapters were added to allow multiplexing of the sequencing libraries. Lastly, cDNA derived from ribosomal RNA was depleted. For purification of the cDNA libraries, AMPure XP beads (Beckman Coulter, USA) were used instead of the recommended NucleoMag NGS Clean-up and Size Select beads (Macherey-Nagel, Germany). The Bioanalyzer High Sensitivity DNA assay with the 2100 Bioanalyzer system (Agilent Technologies, Inc., USA) was used to assess the qualities of the cDNA libraries. The concentrations of the final cDNA libraries were assessed using the 7500 Real-Time PCR System (Applied Biosystems, USA) with the KAPA Library Quantification Kits – Complete kit (ABI Prism) (Kapa Biosystems, USA). Paired-end sequencing (2 x 100 bp) was performed on a NovaSeq 6000 instrument using the NovaSeq 6000 S4 Reagent Kit v1.5 (200 cycles) (Illumina, USA).

### Alignment and normalisation

The quality assessment and alignment of the sequenced reads were performed as previously described [3]. Briefly, the output files from the sequencing instrument were converted to FASTQ-files using *bcl2fastq2* (Illumina, USA). The quality of the sequenced reads was assessed using *FastQC* [18]. To ensure proper alignment of the reads, the adapter sequences, consecutive stretches of low-quality bases (Q < 30) at the 5’ and 3’ termini, and reads shorter than 20 bp were removed using *AdapterRemoval* version 2.3.2 [19]. The reads were aligned using *Spliced Transcripts Alignment to a Reference* (*STAR*) version 2.5.3a [20]. Default settings were used for the alignment except for two parameters: 1) Reads that aligned to multiple loci were defined as unmapped (--outFilterMultimapNmax 1), and 2) the prediction of unannotated splice junctions was disabled (--alignIntronMax 1). Alignment was based on the human genome assembly GRCh38.p13 (hg38) using FASTA- and GTF-files from the GENCODE consortium release 33 [21]. All gene features in the GENCODE GTF-file, constituting 58,601 genes (coding and non-coding), were included in the analyses unless otherwise stated. Transcripts included in the subsets of protein-coding RNAs, mitochondrial RNAs (mtRNAs), and long non-coding RNAs (lncRNAs) were defined based on gene annotations in the GENCODE consortium release 33 [21]. mtRNAs were defined as RNAs transcribed from the mitochondrial genome. Nuclear transcripts encoding proteins translocated into the mitochondria were considered protein-coding.

The number of reads assigned to each gene from duplicate investigations were summed before normalisation. To account for differences in sequencing depth, the gene counts were reported as moderated log_2_(counts per million (CPM)), where moderated indicates that a small number, proportional to the sequencing depth, was added to all values to avoid taking the log of zero. Graphics were created using the *ggplot2*-package version 3.3.5 [22] in *R* version 4.1.2 [23].

### Assessment of the relative rate of decay

Gene expression is reported as a proportion of the total sequenced reads (CPM). We hypothesise that, if all transcripts decay at equal rates, the global gene expression profiles would remain the same over time, and the relative rank of the transcripts’ expression levels would be constant. However, with different decay rates, the relative amounts of transcripts may shift, whereby global gene expression profiles may change (as illustrated in S1 Fig). We defined the relative decay rate as the slope of the linear mixed effects model of log-transformed expression levels (CPM) for storage times zero, one, seven, 14, and 28 days. The different temperatures, 4°C and 22°C, were investigated separately. The model included random intercepts for each individual to account for inter-individual differences in gene expression. To account for multiple testing, Benjamini-Hochberg (false discovery rate – FDR) correction was performed.Genes with negative slopes, statistically significantly different from zero (FDR < 0.05), were classified as genes with fast decay rates, genes with positive slopes, statistically significantly different from zero (FDR < 0.05), were classified as genes with slow decay rates, and all other genes were classified as having intermediate decay rates.

Gene length was assessed using annotations in the GTF-file from the GENCODE consortium release 33 and calculated as the sum of bases of all exons. GC content was calculated using *BEDTools* version 2.22.1 [24]. Rain cloud plots were generated in *R* using the *ggplot2*-package, *ggdist*-package version 3.2.1 [25], and *gghalves*-package version 0.1.4 [26].

### Principle component analysis

Principal component analysis (PCA) was performed using the *prcomp()* function in *R*. The associations between principal components (PCs) and investigated variables were assessed by simple linear regression using the *plomics*-package version 0.2.0 [27] in *R*. Graphics were generated in *R* using the *ggplot2*-package.

### Differential expression analysis

Before differential expression (DE) analysis, read counts were normalised using the trimmed mean of M values (TMM) method, which corrects for sequencing depth and RNA composition. DE analysis was performed using the *edgeR*-package [28] in *R*, using the quasi-likelihood F-test framework. Storage times were used as the grouping variable, whereas patient ID was used as a blocking variable to account for the paired experimental setup. The different temperatures, 4°C and 22°C, were investigated separately. Gene expression patterns on day zero were used as a reference. An FDR < 5% was used. Venn diagrams were created using the *ggVennDiagram*-package [29] and *ggplot2*-package in *R*.

Additional DE models were defined to examine the effects of excluding genes with low expression or including RIN or DV200 as covariates. Genes with low expression were filtered using the *filterByExpr()* function in the *edgeR*-package in *R*.

## Results

To investigate the effects of tissue storage at different temperatures for extended periods of time before RNA sequencing, we performed WTS of RAA tissue stored at either 4°C or 22°C for zero, one, seven, 14, and 28 days. The median storage time for the reference samples (day zero) was 45 minutes (range: 31–56 minutes).

### RNA integrity and yield of sequencing

The quality of extracted RNA was evaluated in terms of both the RIN and DV200. We observed a gradual decrease in RNA integrity with longer storage times, suggesting a continuous degradation of RNA over time (S2 Fig, S2 and S3 Tables). The degradation of RNA appeared faster at 22°C compared to 4°C.

The WTS yielded a median of ~59 million sequencing reads (range: 22–100 million reads) per sequencing library. No apparent bias in the number of sequenced reads among storage times or temperatures was observed (S3 Fig). One of two replicate investigations on day 14 at 22°C was omitted from the analysis due to suspicion of DNA contamination of the RNA extract.

High correlations between global gene expression levels of replicate investigations were observed (median ρ = 0.87) (S4 Fig).

### Alignment statistics and relative RNA abundance

The alignment statistics were examined to assess the proportion of successfully aligned reads and the abundance of different RNA subtypes. Over time, we observed an increase in the proportion of reads discarded before alignment due to low quality (Q < 30) (S5 Fig). In addition, with increasing storage times, we observed larger proportions of reads that did not align to the human genome and reads aligning to intronic and intergenic regions. On average, 40% of the sequenced reads aligned uniquely to gene-annotated regions (S5 and S6 Figs). All other reads were discarded from the dataset in subsequent analyses.

**Fig 2:**
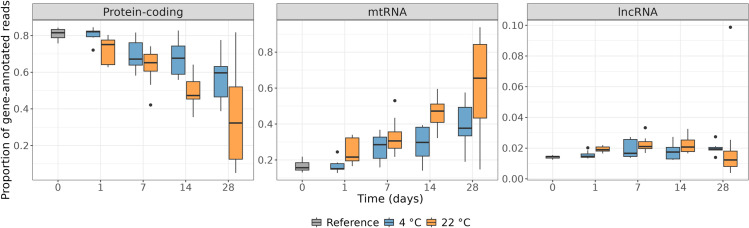
Proportion of protein-coding, mitochondrial (mtRNA), and long non-coding RNAs (lncRNA) of all gene-annotated sequencing reads from tissues stored at varying time intervals and temperatures.

Of the gene-annotated reads, the major constituents were protein-coding, mitochondrial, and long non-coding RNA reads (S7 Fig). The proportion of reads aligning to protein-coding genes decreased with increasing storage time (Fig 2). This pattern was more pronounced at 22°C than 4°C. Conversely, the proportion of mitochondrial reads increased with time and temperature (Fig 2). Lastly, the proportion of reads aligning to long non-coding genes remained relatively stable (Fig 2).

### Differences in decay rates

The gene-wise RNA decay rates were estimated by assessing systematic increases or decreases in gene expression levels over 28 days. Of 58,601 investigated genes, 15,448 (26.4%) genes had slow decay rates at 4°C, whereas 8,431 (14.4%) genes had fast decay rates. At 22°C, 4,462 (7.6%) genes had slow decay rates, whereas 12,648 (21.6%) genes had fast decay rates. As indicated by the overall proportion of mitochondrial and protein-coding transcripts over time, we found that most mitochondrial transcripts had slow decay rates, whereas up to 50% of the protein-coding transcripts had fast decay rates (S8 Fig). The GC content did not have a consistent effect on the decay rate (S9 Fig). However, longer genes were associated with faster decay rates at both 4°C and 22°C (S10 Fig).

### Global gene expression profiles

**Fig 3:**
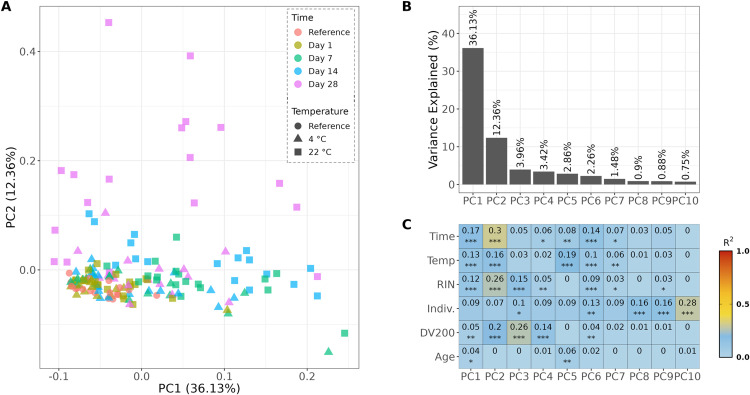
Principal component analysis (PCA) of global gene expression profiles. A) PCA plot of all samples stratified by time and temperature. B) Variance explained by principal components (PCs) 1–10. C) Association analysis of PCs 1–10 and variables. R^2^ = Squared Pearson correlation coefficient, * = p-value < 0.05, ** = p-value < 0.01, *** p-value < 0.0001.

To assess potential sources of variation in the global gene expression profiles, PCA was performed (Fig 3.A-B). Associations between PCs and biological/technical variables were investigated. Storage time and temperature, and the resulting altered RNA integrity, statistically significantly correlated with PC1 and PC2, which explained nearly 50% of the variance (Fig 3.C). To a much lower degree, the variance could be explained by inter-individual differences in gene expression profiles.

### Differential expression analysis

To assess whether the effects of time and temperature were sufficient for interference with common gene expression investigations, we performed a pairwise DE analysis of tissues with different storage times and temperatures. The tissues investigated at day zero were used as references, and all comparisons were performed within individuals. At both temperatures, we observed an increasing number of genes classified as DE with longer storage times (Table 1.A, S11 Fig). At 4°C, a gradual increase in the number of DE genes was observed with time, whereas more than half of the investigated genes were statistically significantly different after day seven at 22°C. Working with cardiac tissue, we investigated whether 41 genes, associated with “Cardiovascular” and “Cardiovascular metabolic” phenotypes from the American College of Medical Genetics and Genomics (ACMG) SF v3.2 list [30], were classified as DE. No cardiac genes were classified as DE after one day of storage (S4 Table). However, after seven days of storage, a significant number of cardiac genes were falsely classified as DE.

**Table 1:** Number of reported differentially expressed genes in paired tissues stored for one, seven, 14, and 28 days at 4°C and 22°C prior to RNA extraction. Pairwise differential expression analysis was performed using day zero as the reference. A) The reported number of differentially expressed genes without including covariates in the model. B) The reported number of differentially expressed genes when including RNA integrity number (RIN) as a covariate. Percentages are of the total number of tested genes (n = 58,601). False discovery rate < 5% was used.

A - No covariates	4°C	22°C
Day 1	**1** (0.002%)	**113** (0.19%)
Day 7	**4,492** (7.67%)	**34,118** (58.22%)
Day 14	**6,993** (11.93%)	**38,879** (66.35%)
Day 28	**15,498** (26.45%)	**37,839** (64.57%)
B - RIN as covariate		
Day 1	**1** (0.002%)	**117** (0.20%)
Day 7	**13** (0.03%)	**5,443** (9.29%)
Day 14	**40** (0.07%)	**11,511** (19.64%)
Day 28	**81** (0.14%)	**2,291** (3.91%)

**Fig 4:**
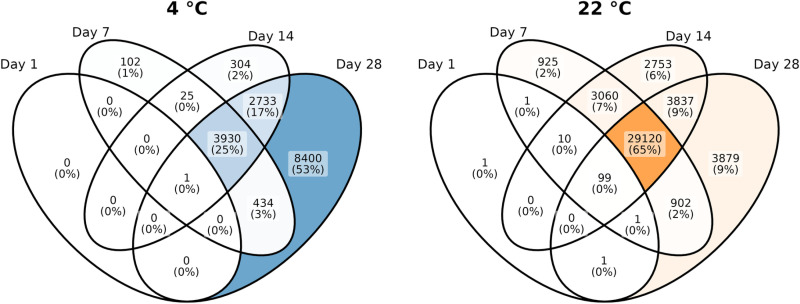
Overlap in genes classified as differentially expressed in paired tissues stored at different time intervals before RNA extraction.

The changes in gene expression profiles appeared systematic over time, as most genes were classified as statistically significantly different at multiple time points (Fig 4). At 4°C, genes classified as DE primarily had fast decay rates (S12 Fig).

### Corrections to minimise the effects of storage conditions

In order to assess the influence of genes with low expression levels, genes with few reads were excluded from the statistical analysis. Exclusion of genes with low expression did not reduce the number of genes reported as differentially expressed (S5 Table).

Storage times and temperatures are not always known, and it may be challenging to include these variables as covariates in the DE model. However, as we observed a decreasing RNA integrity with increasing storage time, we used RIN and DV200 measurements as proxies for storage conditions. Including RIN as a covariate in the DE model drastically reduced the number of reported DE genes in tissues stored for up to 28 days, especially in tissues stored at 4°C (Table 1.B, S13 Fig). While including DV200 as a covariate in the DE model reduced the number of reported DE genes in tissues stored at 4°C, the effect was limited in tissues stored at 22°C (S6 Table).

## Discussion

RNA sequencing is increasingly being used in research and clinical diagnostics and prognostics, so it is essential to understand the effects of pre-analytical variables. This study examined changes in RNA integrity and global gene expression levels in tissues stored for up to 28 days at different temperatures. The time points were chosen to mimic situations where optimal storage of tissues for molecular investigations may not be possible. These conditions may arise in a variety of situations, both clinical and forensic.

As previously reported, we observed a gradual decrease in the integrity of RNA with increasing tissue storage time [11,12]. Additionally, we confirmed that the decrease in RNA integrity was temperature-dependent. Not surprisingly, tissues stored at room temperature were associated with more rapid reductions in RNA integrity than storage at colder temperatures. The RNA degradation is, hence, dependent not only on storage time but, to a large degree, also on storage temperatures. To maintain the integrity of tissues, it is, therefore, important to store tissues at colder temperatures, preferably ≤ 4°C.

Information about the RNA integrity, especially RIN, showed great potential for minimising type I error. By including RIN in the DE analysis, we observed a drastic reduction in the number of DE genes in tissues stored at 4°C. If including measures of RNA integrity in the DE analyses in, e.g., case-control studies, it is, however, important to consider that the distribution of RIN/DV200 scores may mask the underlying biological variation between the investigated groups if RIN and the underlying biological variation are highly correlated.

Through pairwise comparisons of the individual time points with the reference, we found that extended storage time considerably affected the global gene expression patterns. After seven days at 22°C, more than half of the tested genes were falsely classified as DE. Several wrongly classified genes were associated with inherited cardiac diseases, such as *SCN5A*, *KCNQ1*, and *RYR2*, which renders the tissues less suitable for reliable gene expression examinations in relation to cardiac disease. Thus, tissue storage for extended periods may be a significant source of variation in gene expression investigations. As with the investigations of RNA integrity, the number of DE genes is highly dependent on the temperature. Gene expression profiles remained relatively stable for one day, which indicates the utility of tissues stored for up to 24 hours.

This study shows patterns of RNA degradation in tissues where minimal measures are taken to maintain the integrity of the tissue. This was done to elucidate the time-dependent patterns of RNA decay. As observed in previous studies, we expect that more appropriate storage temperatures, such as -80°C, or RNA preservation solutions can reduce RNA decay rates [31–33].

With prolonged tissue storage, we observed a shift in the proportion of mitochondrial and protein-coding RNAs. Mitochondrial transcripts seem better protected against degradation than nuclear-encoded protein-coding transcripts, as protein-coding transcripts appear to have faster decay rates than mitochondrial transcripts. We also observed this pattern in a previous RNA sequencing study, where degraded RNA had an increased proportion of mitochondrial transcripts compared to intact RNA [3]. This pattern was also described using post-mortem tissue, where longer PMIs and increased levels of post-mortem decomposition resulted in increased proportions of mitochondrial transcripts in cardiac tissues [15,34]. This has previously been hypothesised to be due to loss of cytoplasmic RNAs when cell membranes are impaired during tissue degradation [35,36]. We have previously hypothesised that mitochondrial RNA may be protected from degradation by the mitochondrial membranes [3]. Mitochondrial transcripts remain within the mitochondria, whereby the mitochondrial membranes may serve as a barrier against exposure to ribonucleases upon cell degradation. However, this hypothesis still needs to be confirmed by additional research.

We observed relatively stable levels of lncRNAs, which may be explained by their low abundance compared to mitochondrial and protein-coding transcripts. Additionally, the proposed diverse regulatory functions of lncRNAs have been suggested to be mediated through complex secondary and tertiary structures, such as hairpin loops [37,38]. This may increase the stability of lncRNA. However, further studies are needed to elucidate the structural stability of lncRNAs.

A previous study reported that the GC content affects the rate of RNA decay as they observed decreased RNA decay rates with increased GC content [17]. While it is generally accepted that increased GC content is associated with increased DNA stability due to G and C forming three hydrogen bonds as opposed to two hydrogen bonds for A and T [39], we did not observe any association between the GC content and RNA decay rate. This may be explained by the primarily single-stranded structure of RNA molecules. We did, however, find that long transcripts had increased decay rates, which is consistent with results from other studies [40,41].

We added 10 µl isotonic PBS to the tissues during storage to prevent the tissues from drying out. We did this to mimic the conditions of tissues still within the body with compromised blood supply. While our study may not be a suitable model for post-mortem degradation, as additional effects of residual biological processes as a response to organism death and impact of environmental factors are expected, we also anticipate pronounced changes in gene expression levels with extended PMIs. Fluctuations in environmental conditions, such as humidity, would likely also affect RNA decay rates in a clinical or post-mortem setting as higher humidity is associated with accelerated RNA decay rates [42]. Keeping the tissues in a moist environment may have accelerated the degradation processes, whereby the results may not apply to tissues stored in dry environments.

While the paired nature of our experimental setup is a strength of our study, a significant limitation is the sample size. We included nine patients in this study, thus, our results need validation in larger cohorts. Though gene expression profiles remain relatively stable for 24 hours, we observed pronounced changes in gene expression levels after seven days. Further studies are needed to determine when these changes occur between days one and seven. While the clinical relevance for storage times beyond 28 days may be limited, extended storage times may be relevant in a forensic setting. However, with the widespread changes in gene expression profiles observed in our study after 28 days of storage, we expect even longer storage times to show similar results.

In this study, we investigated the effects of time and temperature on the gene expression profiles of RAA tissues. While we expect similar effects in other tissues, further investigations are needed to explore RNA decay rates in a wider range of tissue types. Due to potential differences in mitochondrial gene expression in tissues with varying metabolic activity [43,44], our findings should be validated in other tissue types.

In conclusion, gene expression profiles are relatively stable 24 hours after tissue collection. Temperature is an important determinant of RNA stability. Storing tissues at < 4°C is preferable to maintain the tissues’ integrity. While we found widespread changes in gene expression profiles with storage longer than seven days, it may be possible to counteract some changes by incorporating RIN in the statistical models. However, we don’t recommend using tissues with extended storage at temperatures > 4°C for gene expression analyses.

## Supporting information

S1 Fig Hypothesised relationship between the relative gene expression and tissue storage time with equal or different decay rates. **A)** The relative expression of genes remains constant with equal rates of RNA decay. **B)** With different RNA decay rates, the relative gene expression shifts. In the illustrated example, gene B decays faster than gene A, whereby the relative expression of gene B decreases with time, whereas the relative expression of gene A appears to increase with time. Created with Biorender.com.
(TIF)

S2 Fig Quality scores for RNA extracted from the right atrial appendage tissue with different storage times at 4°C or 22°C. **A)** RNA integrity number (RIN) for RNA extracted from tissues stored for zero, one, seven, 14, and 28 days. **B)** Percentage of RNA fragments >200 nucleotides (DV200) for RNA extracted from tissues stored for zero, one, seven, 14, and 28 days.
(TIF)

S3 Fig Number of sequencing reads passing quality control filter (Q > 30). **A)** Number of sequencing reads passing filter for tissues stored for zero, one, seven, 14, and 28 days prior to RNA extraction. **B)** Number of sequencing reads passing filter for tissues stored at 4°C or 22°C.
(TIF)

S4 Fig Correlation of normalised global gene expression levels in replicate investigations. The Spearman’s correlation coefficient (ρ) is printed in the scatter plot. Correlation analysis for patient P12 on day 14 at 22°C is not shown, as one of two replicates was omitted from subsequent analyses. p < 2.2· 10^-16^ for all comparisons. Abbreviations: CPM = Counts per million.
(TIF)

S5 Fig Alignment statistics of whole transcriptome sequencing of RNA extracted from tissues with varying storage time and temperature. Only reads assigned to the “Mapped reads: Gene-annotated reads” were used in subsequent analyses. QC = Quality control (Sequencing quality Q > 30).
(TIF)

S6 Fig Alignment statistics of whole transcriptome sequencing of RNA extracted from tissues with varying storage times and temperatures. (TIF)

S7 Fig RNA type abundance in whole transcriptome sequencing data from tissues with varying storage times and temperatures. (TIF)

S8 Fig The proportion of protein-coding (n = 19,544), mitochondrial (mtRNA)(n = 37), and long non-coding RNAs (lncRNA)(n = 16,652) with slow, intermediate, and fast decay rates according to the the linear mixed effects model of log-transformed expression levels at 4°C and 22°C. (TIF)

S9 Fig Rain cloud plots of the GC content in genes with slow, intermediate, and fast decay rates at 4°C and 22°C. (TIF)

S10 Fig Rain cloud plots of the gene lengths in genes with slow, intermediate, and fast decay rates at 4°C and 22°C. bp = base pairs. (TIF)

S11 Fig Volcano plots of genes reported as differentially expressed in paired tissues stored at different times and temperatures. Differential expression analysis was performed by pairwise comparison with the reference sample (day 0). **A)** One day of storage at 4°C. **B)** Seven days of storage at 4°C. **C)** 14 days of storage at 4°C. **D)** 28 days of storage at 4°C. **E)** One day of storage at 22°C. **F)** Seven days of storage at 22°C. **G)** 14 days of storage at 22°C. **H)** 28 days of storage at 22°C.
(TIF)

S12 Fig Rates of decay for genes reported as differentially expressed (DE). DE analysis was performed by pairwise comparison with the reference sample (day 0). The total number of reported DE genes for each time point is specified above each bar.
(TIF)

S13 Fig Volcano plots of genes reported as differentially expressed in paired tissues stored at different times and temperatures when the RNA integrity number is included as a covariate. Differential expression analysis was performed by pairwise comparisons with the reference sample (day 0). **A)** One day of storage at 4°C. **B)** Seven days of storage at 4°C. **C)** 14 days of storage at 4°C. **D)** 28 days of storage at 4°C. **E)** One day of storage at 22°C. **F)** Seven days of storage at 22°C. **G)** 14 days of storage at 22°C. **H)** 28 days of storage at 22°C.
(TIF)

S1 Table Descriptive characteristics of the study population. CABG = Coronary artery bypass graft.
(PDF)

S2 Table RNA integrity numbers (RIN) for RNA extracted from right atrial appendage tissue with varying storage times and temperatures. (PDF)

S3 Table Percentage of RNA fragments > 200 nt (DV200) for RNA extracted from right atrial appendage tissue with varying storage times and temperatures. (PDF)

S4 Table Reported differential expression (DE) of genes associated with “cardiovascular” and “cardiovascular metabolic” phenotypes from the American College of Medical Genetics and Genomics (ACMG) SF v3.2 list. Genes were categorised as DE with false discovery rate < 5%.
(PDF)

S5 Table Number of reported differentially expressed genes in paired tissues stored for one, seven, 14, and 28 days at 4°C and 22°C before to RNA extraction when genes with low expression were excluded from the analysis. Pairwise differential expression analysis was performed using day 0 as the reference. Percentages are of the total number of tested genes (n). A false discovery rate < 5% was used.
(PDF)

S6 Table Number of reported differentially expressed genes in paired tissues stored for one, seven, 14, and 28 days at 4°C and 22°C before RNA extraction when correcting for the percentage of RNA fragments > 200 nucleotides. Percentages are of the total number of tested genes (n = 58,601). A false discovery rate < 5% was used.
(PDF)
