# Supplementary figures and images for: RNA degradation patterns in cardiac tissues kept at different time intervals and temperatures before RNA sequencing
Source: PLoS One. 2025 May 15;20(5):e0323786. doi: 10.1371/journal.pone.0323786 (PMC12080774; doi:10.1371/journal.pone.0323786)

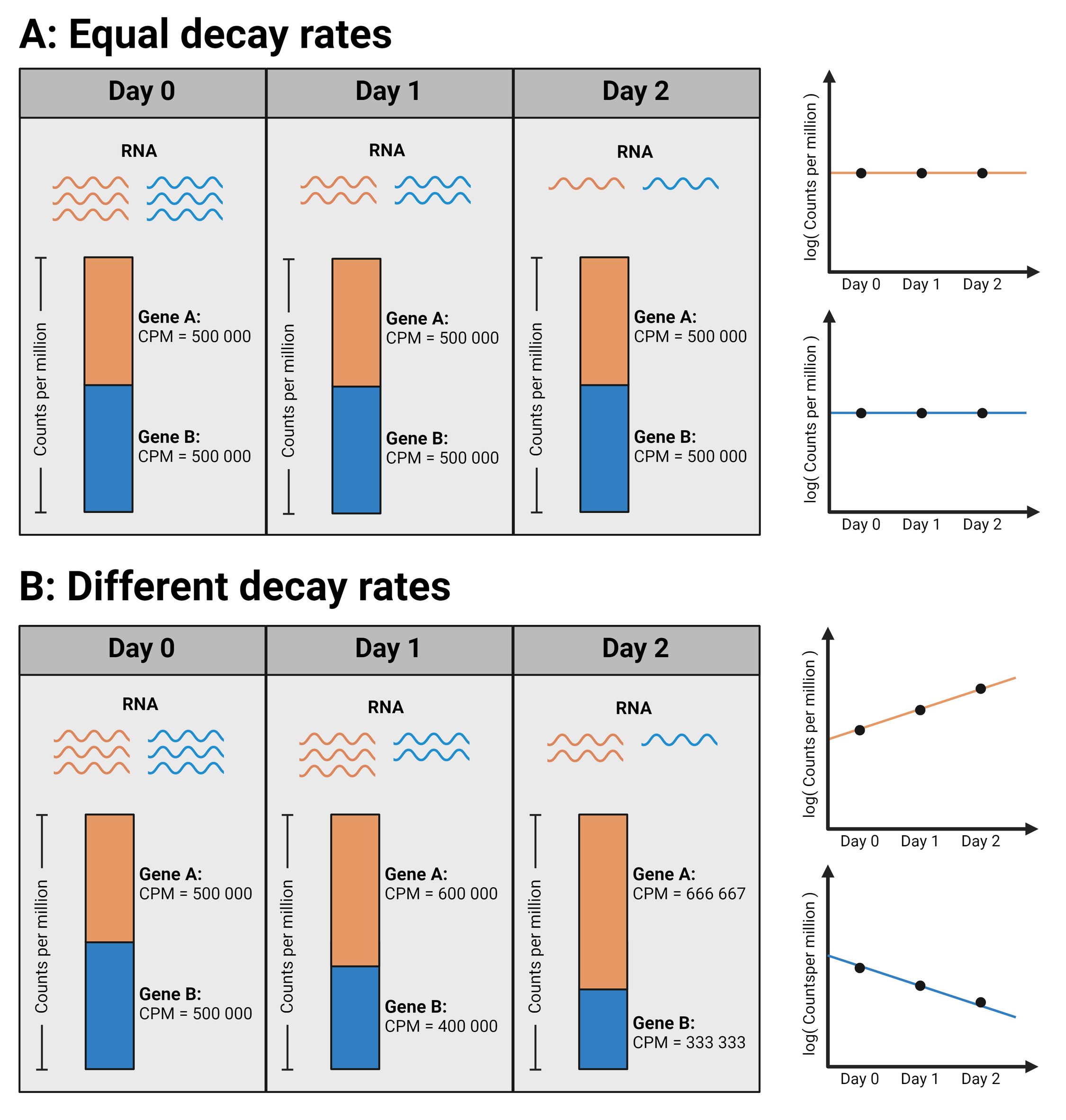

Supplement: S1 Fig — A) The relative expression of genes remains constant with equal rates of RNA decay. B) With different RNA decay rates, the relative gene expression shifts. In the illustrated example, gene B decays faster than gene A, whereby the relative expression of gene B decreases with time, whereas the relative expression of gene A appears to increase with time. Created with Biorender.com. (TIF) [file pone.0323786.s001.tif]

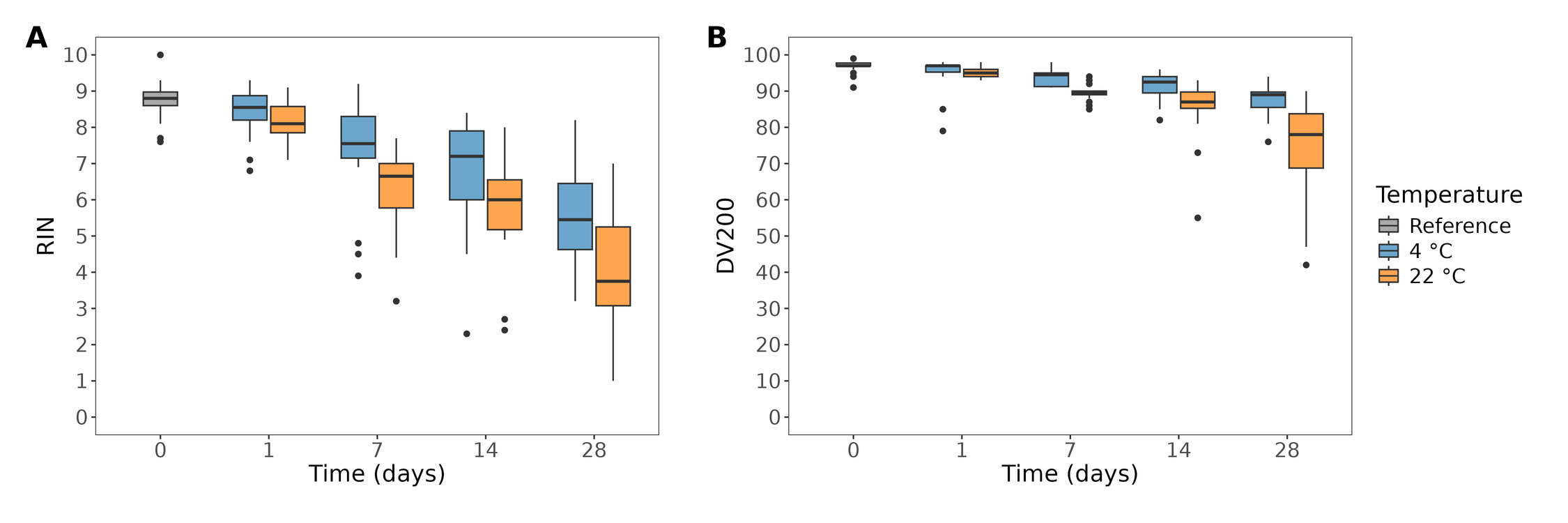

Supplement: S2 Fig — A) RNA integrity number (RIN) for RNA extracted from tissues stored for zero, one, seven, 14, and 28 days. B) Percentage of RNA fragments >200 nucleotides (DV200) for RNA extracted from tissues stored for zero, one, seven, 14, and 28 days. (TIF) [file pone.0323786.s002.tif]

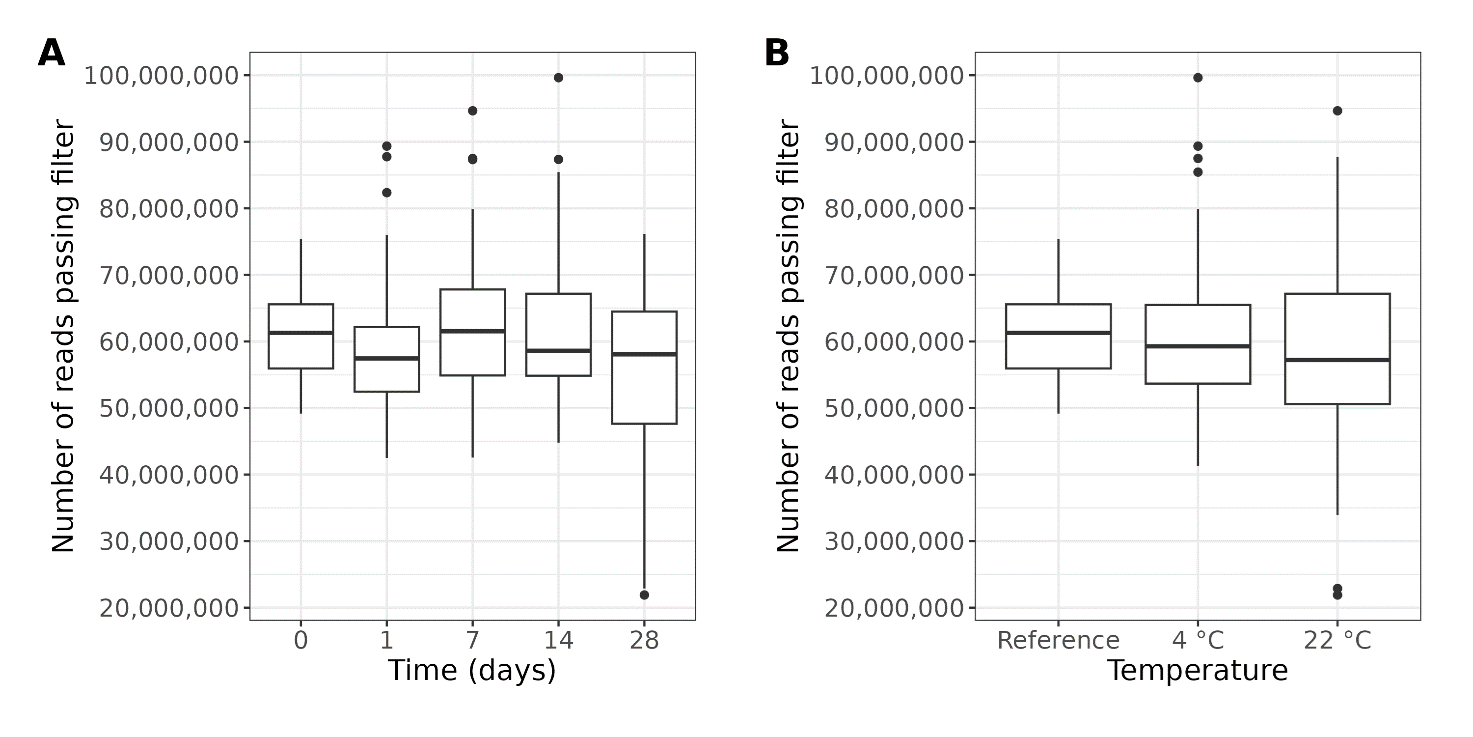

Supplement: S3 Fig — A) Number of sequencing reads passing filter for tissues stored for zero, one, seven, 14, and 28 days prior to RNA extraction. B) Number of sequencing reads passing filter for tissues stored at 4°C or 22°C. (TIF) [file pone.0323786.s003.tif]

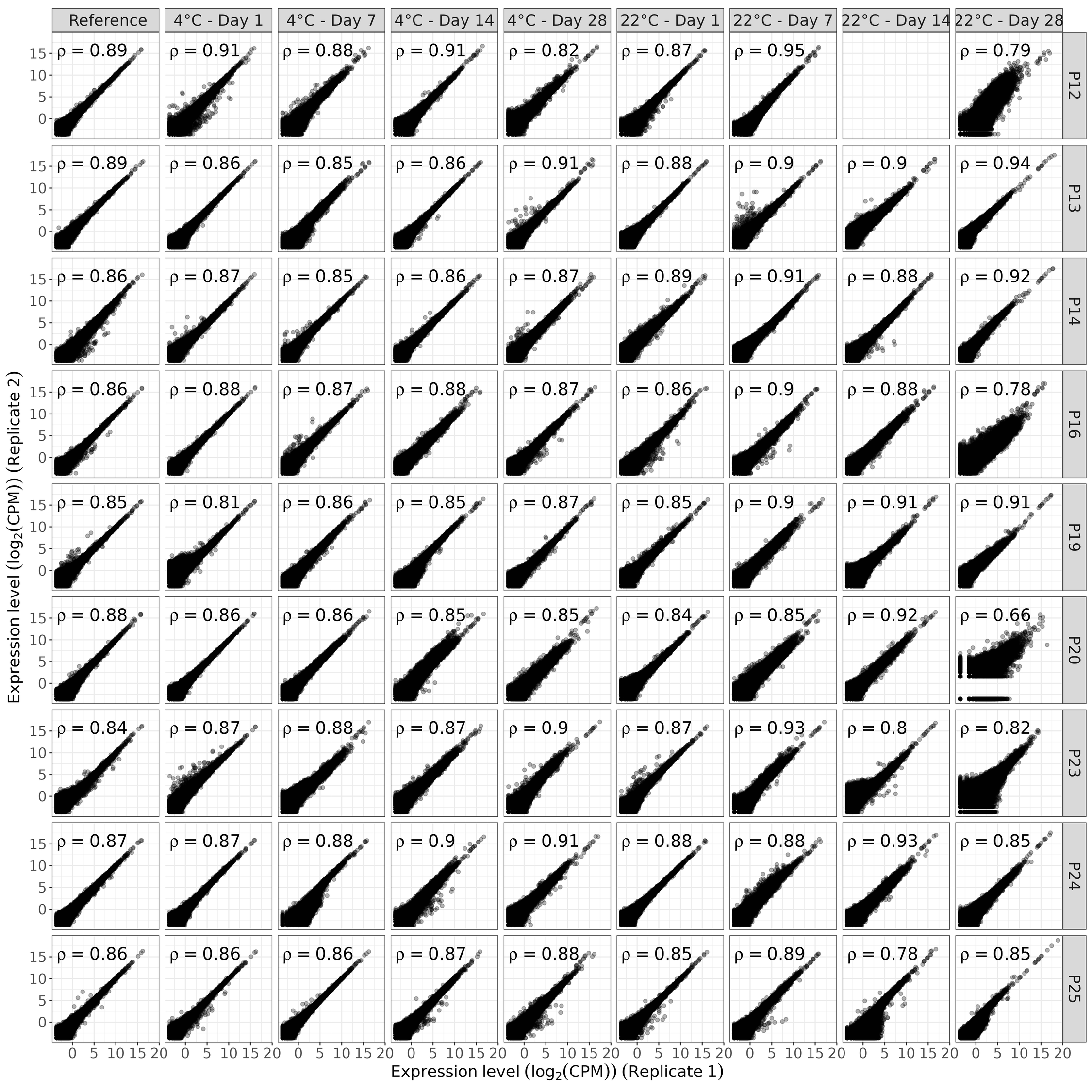

Supplement: S4 Fig — The Spearman’s correlation coefficient (ρ) is printed in the scatter plot. Correlation analysis for patient P12 on day 14 at 22°C is not shown, as one of two replicates was omitted from subsequent analyses. p < 2.2· 10-16 for all comparisons. Abbreviations: CPM = Counts per million. (TIF) [file pone.0323786.s004.tif]

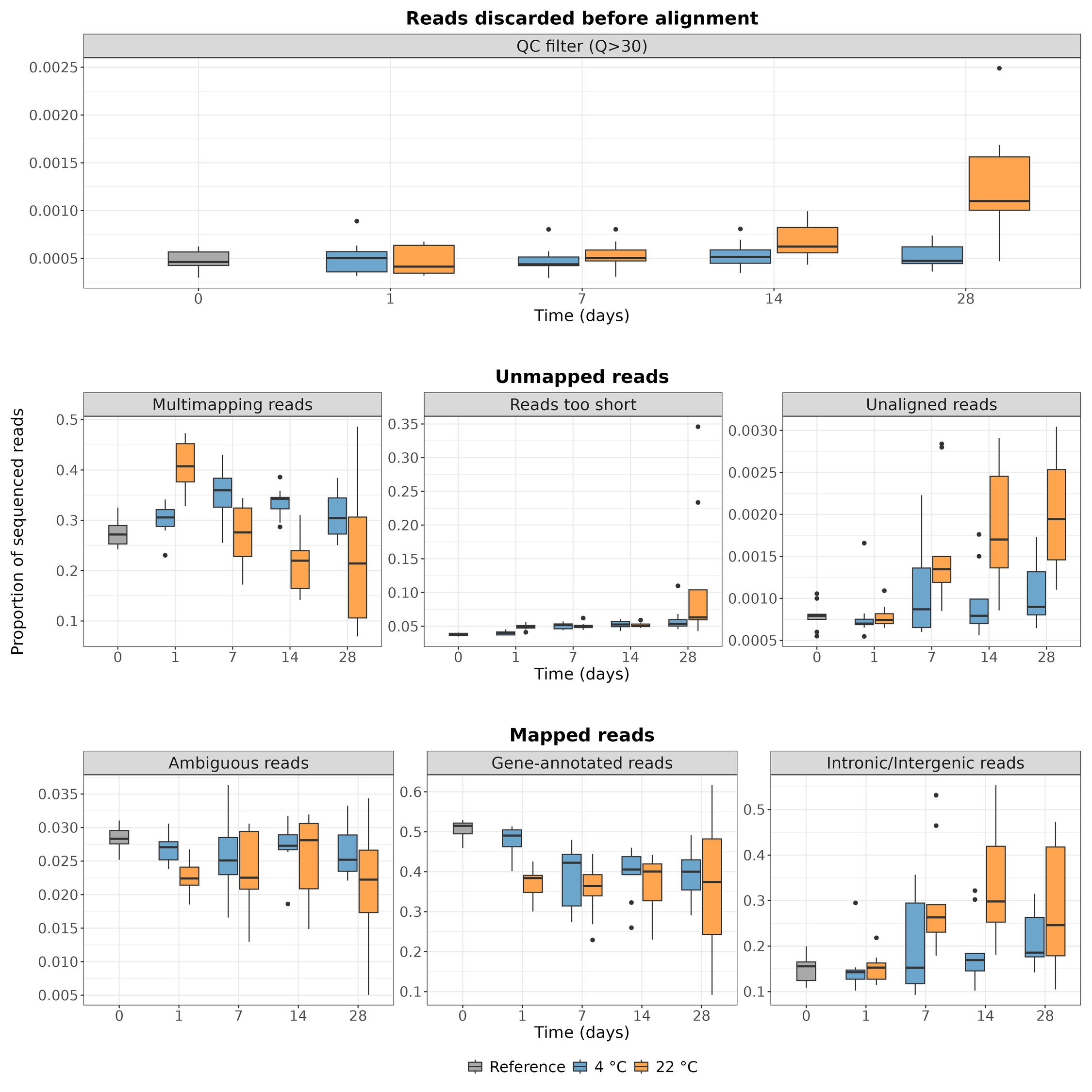

Supplement: S5 Fig — Only reads assigned to the “Mapped reads: Gene-annotated reads” were used in subsequent analyses. QC = Quality control (Sequencing quality Q > 30). (TIF) [file pone.0323786.s005.tif]

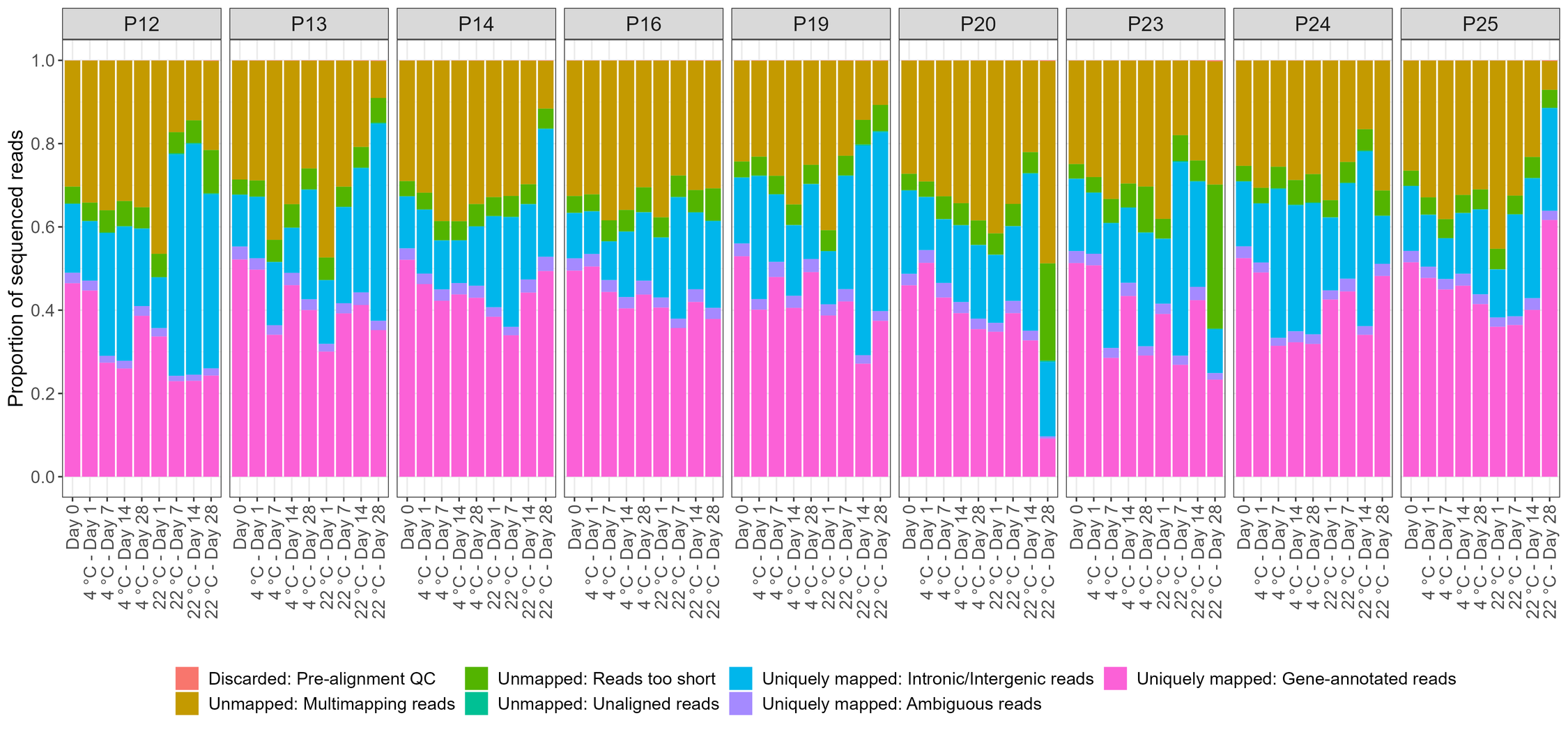

Supplement: S6 Fig — (TIF) [file pone.0323786.s006.tif]

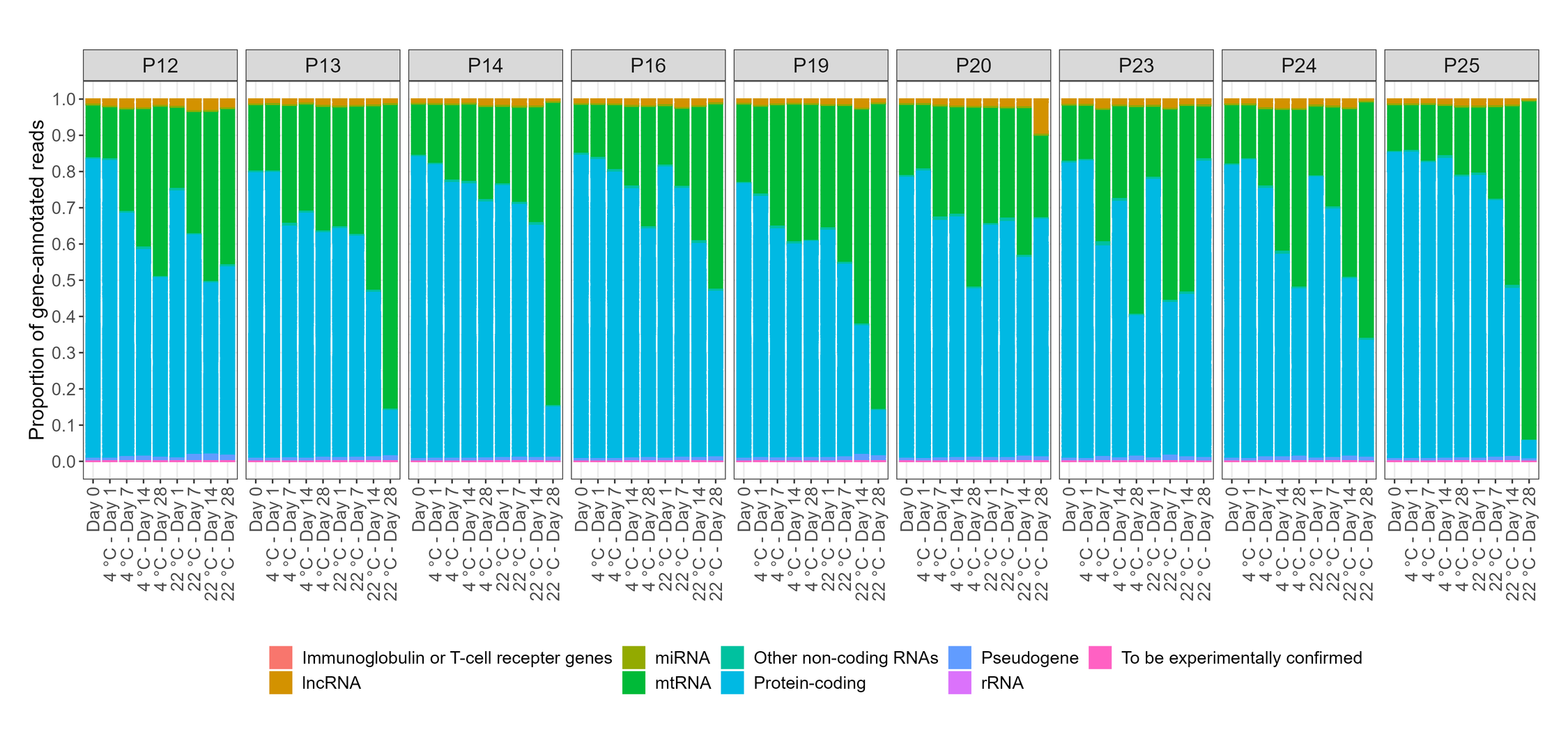

Supplement: S7 Fig — (TIF) [file pone.0323786.s007.tif]

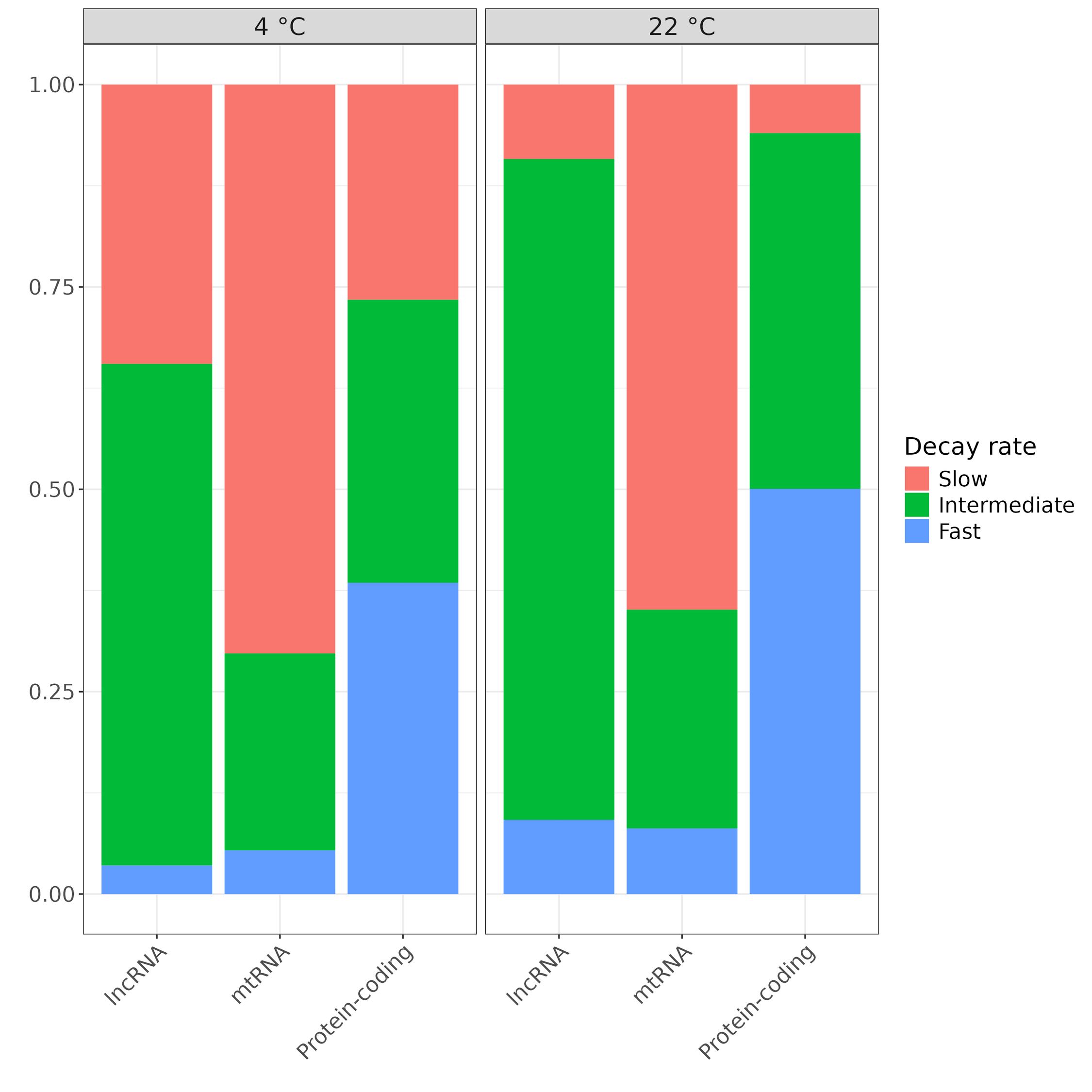

Supplement: S8 Fig — (TIF) [file pone.0323786.s008.tif]

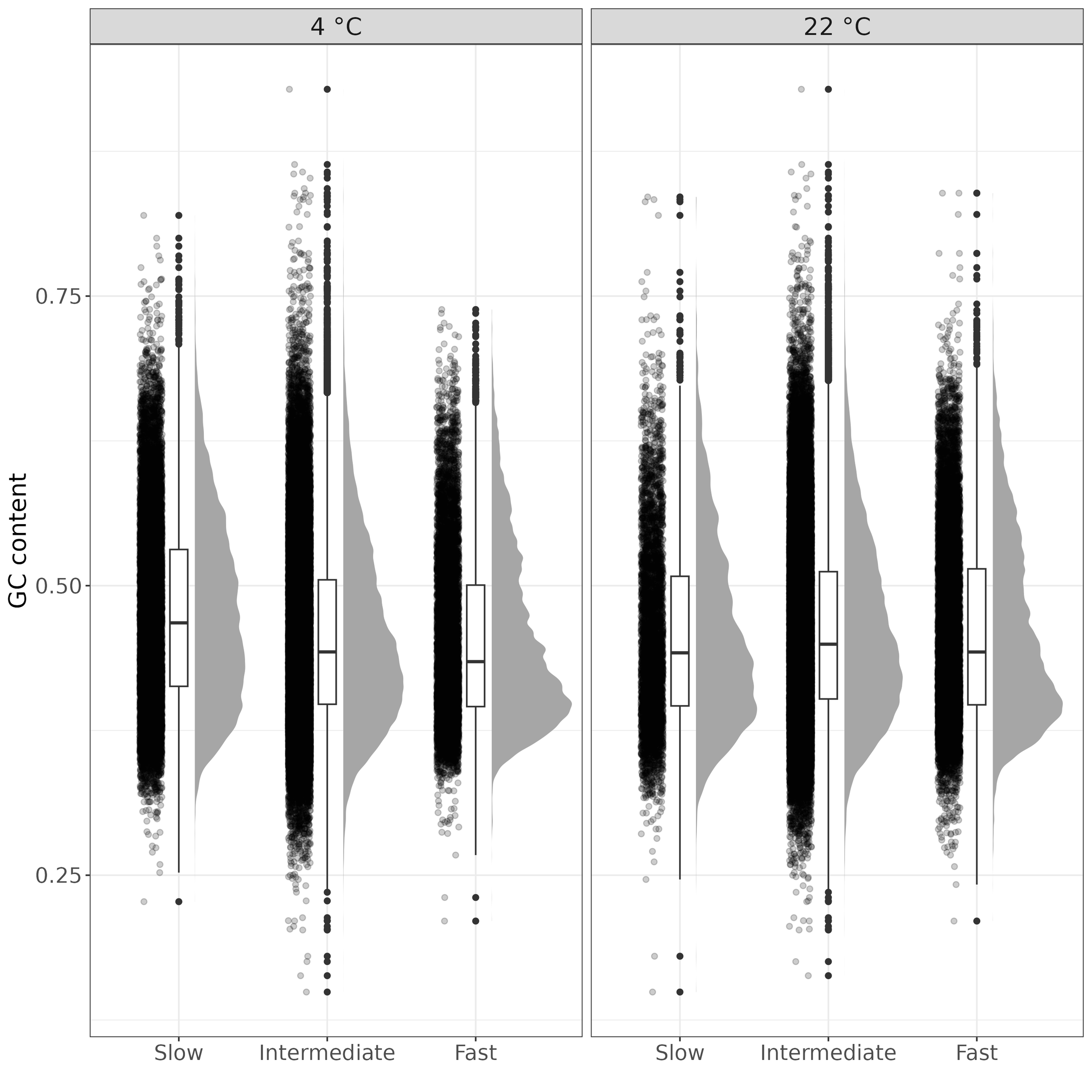

Supplement: S9 Fig — (TIF) [file pone.0323786.s009.tif]

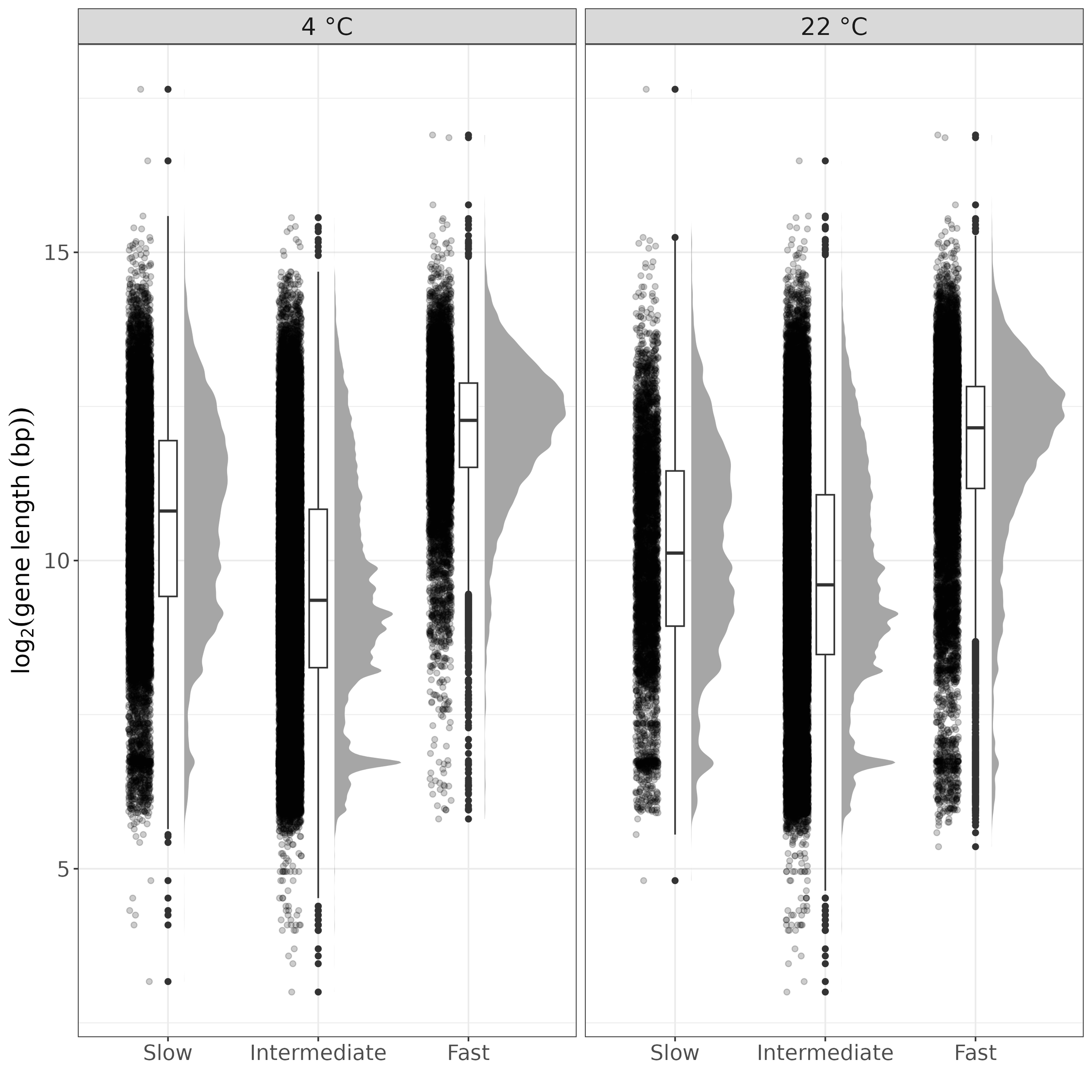

Supplement: S10 Fig — (TIF) [file pone.0323786.s010.tif]

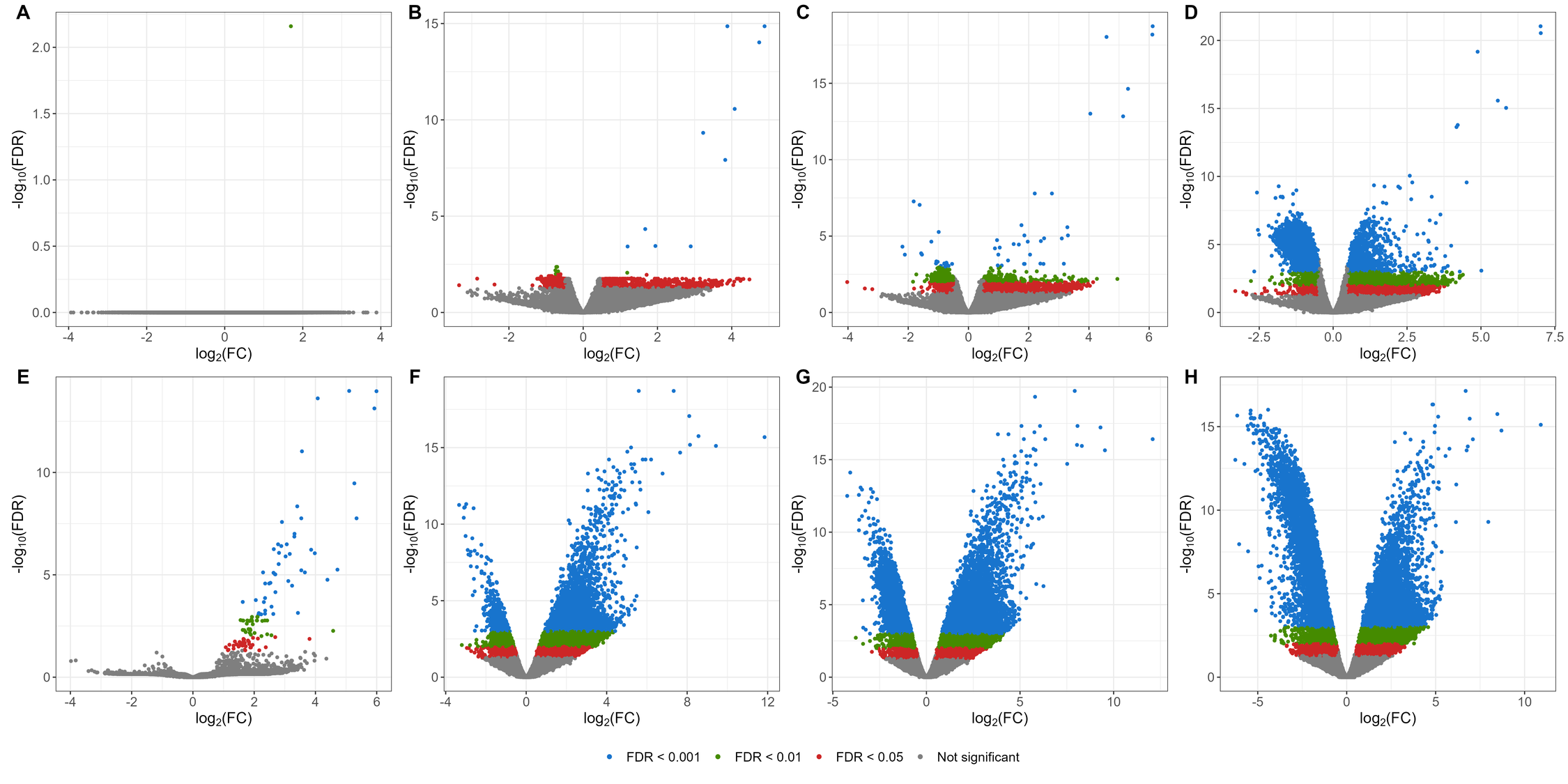

Supplement: S11 Fig — Differential expression analysis was performed by pairwise comparison with the reference sample (day 0). A) One day of storage at 4°C. B) Seven days of storage at 4°C. C) 14 days of storage at 4°C. D) 28 days of storage at 4°C. E) One day of storage at 22°C. F) Seven days of storage at 22°C. G) 14 days of storage at 22°C. H) 28 days of storage at 22°C. (TIF) [file pone.0323786.s011.tif]

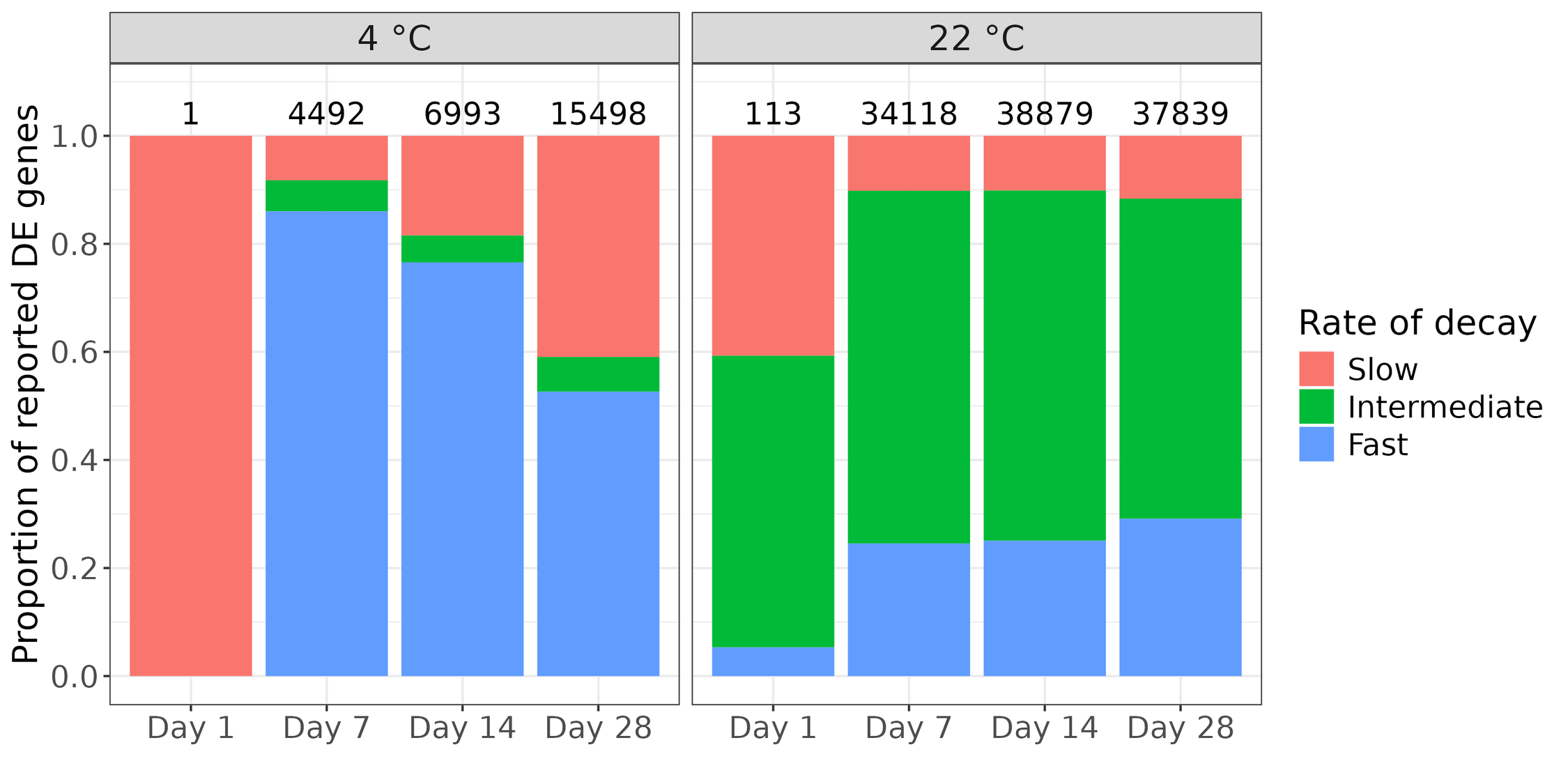

Supplement: S12 Fig — DE analysis was performed by pairwise comparison with the reference sample (day 0). The total number of reported DE genes for each time point is specified above each bar. (TIF) [file pone.0323786.s012.tif]

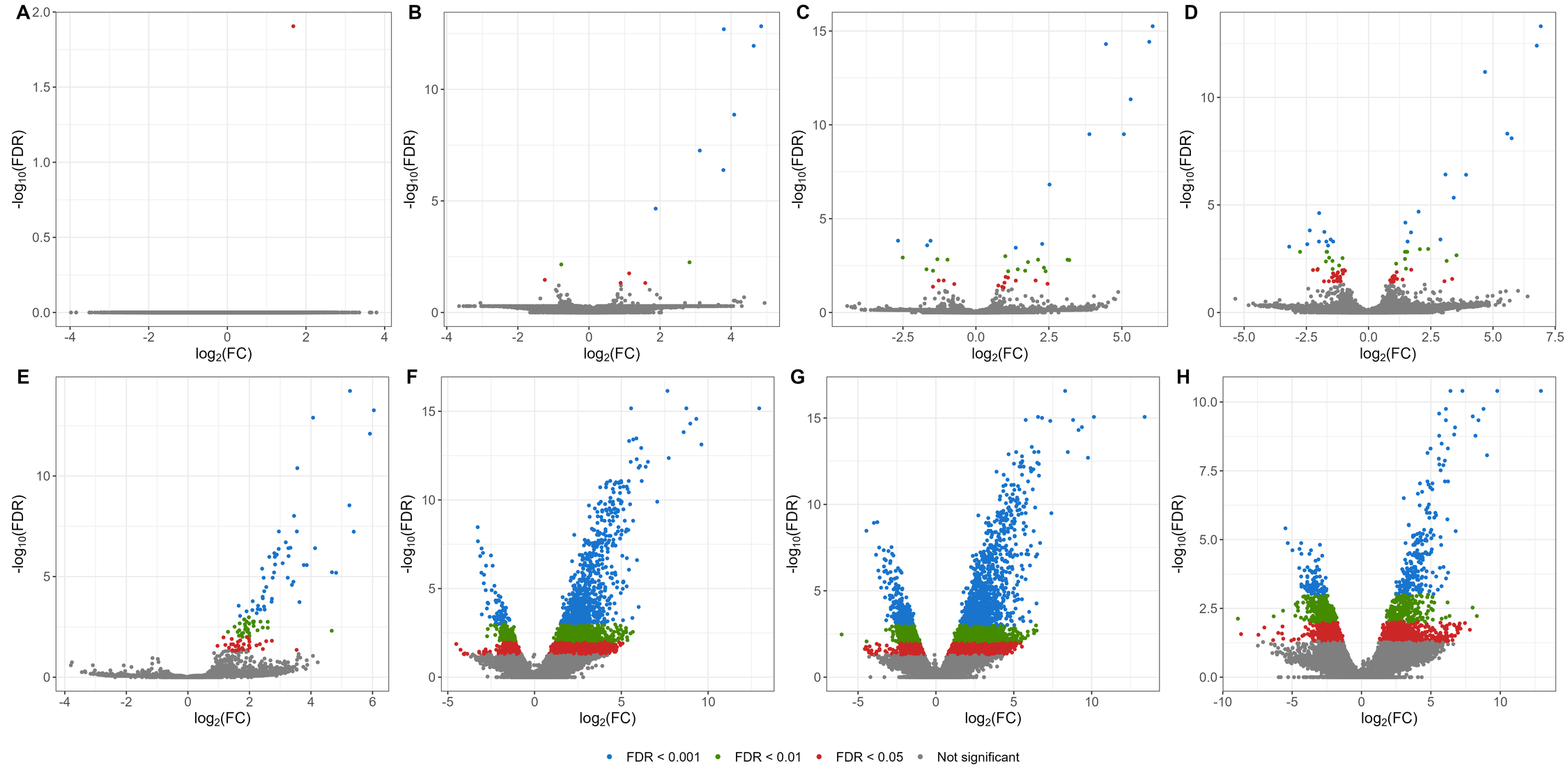

Supplement: S13 Fig — Differential expression analysis was performed by pairwise comparisons with the reference sample (day 0). A) One day of storage at 4°C. B) Seven days of storage at 4°C. C) 14 days of storage at 4°C. D) 28 days of storage at 4°C. E) One day of storage at 22°C. F) Seven days of storage at 22°C. G) 14 days of storage at 22°C. H) 28 days of storage at 22°C. (TIF) [file pone.0323786.s013.tif]
