# Supplementary material for: RNA degradation patterns in cardiac tissues kept at different time intervals and temperatures before RNA sequencing
Source: PLoS One. 2025 May 15;20(5):e0323786. doi: 10.1371/journal.pone.0323786 (PMC12080774; doi:10.1371/journal.pone.0323786)
Supplement: S1 Table — CABG = Coronary artery bypass graft. (PDF) [file pone.0323786.s014.pdf]

S1 Table: Descriptive characteristics of the study population. *CABG = Coronary artery bypass graft.*

**Characteristics**	**n = 9**
Male sex, n (%)	9 (100 %)
Median age at time of tissue collection, years (range)	64 (56-76)
Type of surgery performed*, n (%)	
CABG	6 (66.7 %)
Valve repair/replacement	2 (22.2 %)
Aneurysm	2 (22.2 %)
History of atrial fibrillation, n (%)	
Before surgery	1 (11.1 %)
During surgery	1 (11.1 %)
After surgery	5 (55.6 %)

* One patient underwent both aneurysm and valve repair/replacement surgery.
