# Supplementary material for: RNA degradation patterns in cardiac tissues kept at different time intervals and temperatures before RNA sequencing
Source: PLoS One. 2025 May 15;20(5):e0323786. doi: 10.1371/journal.pone.0323786 (PMC12080774; doi:10.1371/journal.pone.0323786)
Supplement: S2 Table — (PDF) [file pone.0323786.s015.pdf]

S2 Table: RNA integrity numbers (RIN) for RNA extracted from right atrial appendage tissue with varying storage times and temperatures.

Patient ID	Day 0	4 °C				22 °C			
		Day 1	Day 7	Day 14	Day 28	Day 1	Day 7	Day 14	Day 28
P12 – Rep 1	7.6	8.7	8.4	7.9	6.3	8.3	7.3	6	6.6
P12 – Rep 2	9	9.1	8.5	NA	6.3	8	6.9	2.4*	3
P13 – Rep 1	9.2	8.2	7.1	6.7	3.2	8.5	6.6	4.9	4.7
P13 – Rep 2	8.9	8.5	9.2	6.7	5.1	8.6	7.5	5.9	3.5
P14 – Rep 1	8.7	7.6	7.3	8.4	5.1	7.3	7.2	6.6	3.7
P14 – Rep 2	8.6	7.1	7.6	8.2	6.7	7.1	7	6.8	2.3
P16 – Rep 1	8.8	8.5	8.3	7.7	4.5	8.1	7	5	5.3
P16 – Rep 2	8.7	8.6	8	6.4	5.2	8.1	6.3	5.9	5.4
P19 – Rep 1	8.6	8	6.9	7.2	6.5	8.1	5.6	2.7	5.1
P19 – Rep 2	7.7	6.8	8	7.5	5.7	7.6	6.5	5.4	5.1
P20 – Rep 1	8.9	8.7	3.9	5	6.6	8.1	6.4	6.7	1
P20 – Rep 2	8.1	8.2	4.8	8	3.2	7.8	5.3	6	1.1
P23 – Rep 1	9	8.9	7.4	6	4.7	8.5	3.2	6.2	3.5
P23 – Rep 2	8.4	8.4	4.5	4.8	4.6	8.7	4.8	5.1	1.1
P24 – Rep 1	8.8	9	7.3	2.3	3.6	8.8	4.4	6	6.6
P24 – Rep 2	8.9	9.3	7.5	4.5	5.8	8.8	6.7	6.4	7
P25 – Rep 1	9.3	8.8	8.7	7.6	8.2	7.6	7	6.6	3.3
P25 – Rep 2	10	9	8.3	8.4	7.3	9.1	7.7	8	3.8

Abbreviations: NA = Not available (Bioanalyzer unable to calculate RIN), Rep = replicate, *RNA sample with suspected DNA contamination.
