# Supplementary material for: RNA degradation patterns in cardiac tissues kept at different time intervals and temperatures before RNA sequencing
Source: PLoS One. 2025 May 15;20(5):e0323786. doi: 10.1371/journal.pone.0323786 (PMC12080774; doi:10.1371/journal.pone.0323786)
Supplement: S3 Table — (PDF) [file pone.0323786.s016.pdf]

S3 Table: Percentage of RNA fragments > 200 nt (DV200) for RNA extracted from right atrial appendage tissue with varying storage times and temperatures.

Patient ID	Day 0	4 °C				22 °C			
		Day 1	Day 7	Day 14	Day 28	Day 1	Day 7	Day 14	Day 28
P12 – Rep 1	97	94	95	93	90	93	89	86	78
P12 – Rep 2	98	97	93	93	90	94	90	55*	47
P13 – Rep 1	97	96	95	85	83	96	89	90	78
P13 – Rep 2	97	97	95	88	81	96	90	90	68
P14 – Rep 1	97	94	91	96	93	93	92	88	84
P14 – Rep 2	97	85	91	96	89	95	94	88	90
P16 – Rep 1	97	96	96	82	76	96	89	93	81
P16 – Rep 2	99	97	95	92	87	96	89	86	83
P19 – Rep 1	97	95	92	94	89	93	86	82	80
P19 – Rep 2	91	79	95	94	85	93	89	73	77
P20 – Rep 1	95	96	91	89	88	95	88	86	54
P20 – Rep 2	94	97	94	96	85	95	90	85	49
P23 – Rep 1	97	97	93	89	89	96	85	91	71
P23 – Rep 2	96	97	91	91	89	94	87	81	42
P24 – Rep 1	97	98	91	91	87	97	90	89	87
P24 – Rep 2	98	98	97	94	89	98	90	87	85
P25 – Rep 1	98	97	98	92	94	95	90	87	77
P25 – Rep 2	98	97	97	96	92	95	93	92	84

Rep = replicate, *RNA sample with suspected DNA contamination.
