# Supplementary material for: RNA degradation patterns in cardiac tissues kept at different time intervals and temperatures before RNA sequencing
Source: PLoS One. 2025 May 15;20(5):e0323786. doi: 10.1371/journal.pone.0323786 (PMC12080774; doi:10.1371/journal.pone.0323786)
Supplement: S4 Table — Genes were categorised as DE with false discovery rate < 5%. (PDF) [file pone.0323786.s017.pdf]

S4 Table: Reported differential expression (DE) of genes associated with "cardiovascular" and "cardiovascular metabolic" phenotypes from the American College of Medical Genetics and Genomics (ACMG) SF v3.2 list. *Genes were categorised as DE with false discovery rate < 5 %.*

Gene	Phenotype	4 °C				22 °C			
		DE at day 1	DE at day 7	DE at day 14	DE at day 28	DE at day 1	DE at day 7	DE at day 14	DE at day 28
ACTA2	Familial TAA				X		X	X	X
ACTC1	HCM			X	X		X	X	X
APOB	FH		X	X	X		X	X	X
BAG3	DCM & MFM		X	X	X				
CALM1	LQTS & CPVT		X	X	X		X	X	X
CALM2	LQTS & CPVT				X		X	X	X
CALM3	LQTS & CPVT				X		X	X	X
CASQ2	CPVT			X	X		X	X	X
COL3A1	Ehlers-Danlos syndrome						X	X	X
DES	DCM & MFM			X	X		X	X	X
DSC2	ARVC		X	X	X		X	X	X
DSG2	ARVC		X	X	X		X	X	X
DSP	ARVC & DCM			X	X		X	X	X
FBN1	Marfan syndrome						X	X	X
FLNC	DCM & MFM & HCM			X	X		X	X	X
GLA	Fabry disease				X				X
KCNH2	LQTS			X	X		X	X	X
KCNQ1	LQTS			X	X		X	X	X
LDLR	FH						X	X	
LMNA	DCM								
MYBPC3	HCM				X		X	X	X
MYH11	Familial TAA							X	X
MYH7	HCM				X		X	X	X
MYL2	DCM			X	X		X	X	X
MYL3	HCM				X			X	X
PCSK9	FH				X		X	X	X
PKP2	ARVC		X	X	X		X	X	X
PRKAG2	HCM		X	X	X		X	X	X
RBM20	DCM			X	X		X	X	X
RYR2	CPVT			X	X		X	X	X
SCN5A	LQTS & BrS & DCM			X	X		X	X	X
SMAD3	Loeys-Dietz syndrome				X				
TGFBR1	Loeys-Dietz syndrome				X				
TGFBR2	Loeys-Dietz syndrome				X		X	X	X
TMEM43	ARVC				X		X	X	X
TNNC1	DCM			X	X		X	X	X
TNNI3	HCM				X		X	X	X
TNNT2	DCM & HCM						X	X	X
TPM1	HCM				X		X	X	X
TRDN	CPVT & LQTS			X	X		X	X	X
TTN	DCM				X		X	X	X

ARVC = Arrhythmogenic right ventricular cardiomyopathy; BrS = Brugada syndrome; CPVT = Catecholaminergic polymorphic ventricular tachycardia; DCM = Dilated cardiomyopathy; FH = Familial hypercholesterolemia, HCM = Hypertrophic cardiomyopathy; LQTS = Long QT syndrome; MFM = Myofibrillar myopathy; TAA = thoracic aortic aneurysm.
