# Supplementary material for: RNA degradation patterns in cardiac tissues kept at different time intervals and temperatures before RNA sequencing
Source: PLoS One. 2025 May 15;20(5):e0323786. doi: 10.1371/journal.pone.0323786 (PMC12080774; doi:10.1371/journal.pone.0323786)
Supplement: S5 Table — Pairwise differential expression analysis was performed using day 0 as the reference. Percentages are of the total number of tested genes (n). A false discovery rate < 5% was used. (PDF) [file pone.0323786.s018.pdf]

S5 Table: Number of reported differentially expressed genes in paired tissues stored for 1, 7, 14, and 28 days at 4 °C and 22 °C before to RNA extraction when genes with low expression were excluded from the analysis. *Pairwise differential expression analysis was performed using day 0 as the reference. Percentages are of the total number of tested genes (n). A false discovery rate < 5% was used.*

	**4 °C** *n = 39,786*	**22 °C** *n = 45,280*
**Day 1**	**1** (0.003 %)	**106** (0.23 %)
**Day 7**	**1,885** (4.74 %)	**30,546** (67.46 %)
**Day 14**	**6,958** (17.49 %)	**34,419** (76.01 %)
**Day 28**	**17,005** (42.74 %)	**33,749** (74.53 %)
