# Supplementary material for: RNA degradation patterns in cardiac tissues kept at different time intervals and temperatures before RNA sequencing
Source: PLoS One. 2025 May 15;20(5):e0323786. doi: 10.1371/journal.pone.0323786 (PMC12080774; doi:10.1371/journal.pone.0323786)
Supplement: S6 Table — Percentages are of the total number of tested genes (n = 58,601). A false discovery rate < 5% was used. (PDF) [file pone.0323786.s019.pdf]

S6 Table: Number of reported differentially expressed genes in paired tissues stored for 1, 7, 14, and 28 days at 4 °C and 22 °C before RNA extraction when correcting for the percentage of RNA fragments > 200 nucleotides.

Percentages are of the total number of tested genes (n = 58,601). A false discovery rate < 5% was used.

	4 °C	22 °C
**Day 1**	**1** (0.002 %)	**137** (0.24 %)
**Day 7**	**22** (0.04 %)	**34,645** (59.12 %)
**Day 14**	**102** (0.17 %)	**37,143** (63.39 %)
**Day 28**	**470** (0.80 %)	**25,678** (43.82 %)
